# Characterization of dislocations in germanium layers grown on (011)- and (111)-oriented silicon by coplanar and noncoplanar X-ray diffraction^1^

**DOI:** 10.1107/S1600576715005397

**Published:** 2015-04-16

**Authors:** Andrei Benediktovitch, Alexei Zhylik, Tatjana Ulyanenkova, Maksym Myronov, Alex Ulyanenkov

**Affiliations:** a Department of Theoretical Physics, Belarusian State University, Nezavisimosti Avenue 4, Minsk, Belarus; b Rigaku Europe SE, Am Hardtwald 11, Ettlingen, Germany; c Department of Physics, The University of Warwick, Coventry, UK

**Keywords:** strained germanium, silicon, complementary metal-oxide semiconductors, noncoplanar X-ray diffraction

## Abstract

Strained germanium grown on silicon with nonstandard surface orientations like (011) or (111) is a promising material for various semiconductor applications, for example complementary metal-oxide semiconductor transistors. However, because of the large mismatch between the lattice constants of silicon and germanium, the growth of such systems is challenged by nucleation and propagation of threading and misfit dislocations that degrade the electrical properties. To analyze the dislocation microstructure of Ge films on Si(011) and Si(111), a set of reciprocal space maps and profiles measured in noncoplanar geometry was collected. To process the data, the approach proposed by Kaganer, Köhler, Schmidbauer, Opitz & Jenichen [*Phys. Rev. B*, (1997 ▶), **55**, 1793–1810] has been generalized to an arbitrary surface orientation, arbitrary dislocation line direction and noncoplanar measurement scheme.

## Abstract 2

The generalization of the theoretical approach suggested by Kaganer *et al.* [*Phys. Rev. B*, (1997 ▶), **55**, 1793–1810] to an arbitrary surface orientation, arbitrary dislocation line direction and noncoplanar measurement scheme was developed. It was applied to study the dislocation microstructure of Ge films on Si(011) and Si(111) based on a set of reciprocal space maps and profiles measured in noncoplanar geometry.

## 1. Introduction   

With the downscaling of today’s technology to the nanometre level, to create silicon-based complementary metal-oxide semiconductor (CMOS) transistors it becomes necessary to look for alternative channel materials, strain configurations and crystallographic orientations to realize the full potential of the semiconductor band structure, required to obtain the highest electron and hole mobility channels (Takagi *et al.*, 2008 ▶). Strained germanium appears to be one of the most promising alternative channel materials owing to both its intrinsically higher electron and hole mobility values and its compatibility with existing CMOS fabrication techniques (Dobbie *et al.*, 2012 ▶; Myronov *et al.*, 2014 ▶). In addition to strain, further improvements of the device performance can be made by using nonstandard surface orientations such as (011) and (111) to fully exploit the properties of the Ge band structure. High mobility electron and hole channel transistors have already been predicted and demonstrated by several groups using Ge substrates with different crystallographic orientations and strain (Ritenour *et al.*, 2007 ▶; Zimmerman *et al.*, 2006 ▶; Kuzum *et al.*, 2009 ▶; Shang *et al.*, 2003 ▶; Maikap *et al.*, 2007 ▶; Nishimura *et al.*, 2010 ▶; Chui *et al.*, 2002 ▶; Low *et al.*, 2004 ▶). However, because of the large mismatch between the lattice constants of silicon and germanium, the growth of such systems is challenged by nucleation and propagation of threading and misfit dislocations that degrade the electrical properties.

To characterize thin films, a variety of techniques are used, such as transmission electron microscopy, Auger electron spectroscopy, high-resolution X-ray diffraction (HRXRD), X-ray reflectometry *etc*. X-ray characterization techniques are favorable owing to their nondestructive nature and good matching of X-ray wavelength to the atomic scale of modern semiconductor devices. HRXRD is a suitable tool for nondestructive investigation of multilayer structures: the peak position delivers the lattice parameters connected with composition and strain while the peak shape is conditioned by layer thickness and defects present in the sample, the dislocations playing a decisive role in peak profile formation (Benediktovitch, Feranchuk & Ulyanenkov, 2014 ▶).

A theoretical approach for calculation of the diffracted X-ray intensity distribution from epitaxial layers possessing dislocations was developed by Kaganer *et al.* (1997 ▶) and successfully applied to a number of systems (Kaganer *et al.*, 2006*b* ▶; Benediktovich *et al.*, 2011 ▶; Kopp, Kaganer, Baidakova *et al.*, 2014 ▶). The peak shape is sensitive to the reflection used, the measurement geometry, the type of dislocations, their density and their degree of correlation. It was shown that from a set of peaks measured for a number of reflections one can get reliable information about the dislocation type (Kaganer *et al.*, 2006*b* ▶). The underlaying idea is closely connected to the method of full-profile analysis in powder diffractometry: given a set of profiles for different reflections *hkl*, the fitting of the whole set enables the determination of the defect parameters (Ribarik & Ungar, 2010 ▶; Scardi & Leoni, 2002 ▶). The reason is that different defect types produce peak broadening that changes in a different way with *hkl* and, consequently, that enables one to disentangle the contribution from a distinct defect type. For example, the dependence of dislocation-induced broadening on *hkl* is governed by the dislocation contrast factor 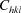, which is specific for each dislocation type, thus enabling one to find the dislocation type from the measured powder profile (Leoni *et al.*, 2007 ▶; Ungár *et al.*, 2001 ▶). A similar approach can be used for treatment of diffraction data measured in HRXRD mode from films containing dislocations. However, in this case one has to modify significantly the underlying theory. In particular, the dislocation contrast factor should be replaced with the dislocation contrast tensor (Benediktovitch, Feranchuk & Ulyanenkov, 2014 ▶), and special attention should be payed to the way that the diffraction signal is collected (Kaganer *et al.*, 2005 ▶). The quantities of interest can be calculated on the basis of the approach presented by Kaganer *et al.* (1997 ▶). However, for each sample normal orientation and dislocation line direction the expressions for intensity distribution should be derived again, accounting for specifics of the geometry. In the current paper we propose a general formalism to treat an arbitrary case of surface orientation and dislocation line direction; also, peculiarities of the application to noncoplanar measurements are discussed.

We have measured a series of reciprocal space maps (RSMs) and profiles for a number of reflections in coplanar as well as noncoplanar measurement geometries to get a consistent data set for analysis of dislocation microstructure. The noncoplanar measurement geometry was achieved by rotations of the detector arm around two orthogonal axes (Rigaku SmartLab diffractometer) without tilting the sample. The developed formalism is applied to the measured data set to obtain information about the dislocation ensemble.

## 2. Sample growth and measurement   

### 2.1. Sample growth   

The epitaxial Ge layers investigated here were grown on 100 mm (111)- and (110)-oriented Si substrates by reduced pressure chemical vapor deposition in an ASM Epsilon 2000 using a GeH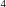 standard as a gaseous precursor diluted in H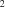 carrier gas. All used wafers were initially baked at 1423 K for 2 min in H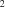 in order to desorb any native oxide on the Si substrates prior to epitaxial deposition. To grow the Ge layer, a fixed GeH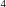 precursor flow rate and chamber pressure of 100 Torr were used (1 Torr = 133.3 Pa), in such a way that the GeH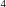 partial pressure was held constant at 75 mTorr for both low-temperature (LT) and high-temperature (HT) stages. It was shown earlier by Shah *et al.* (2011 ▶) that a similar approach is capable of producing high-quality relaxed Ge buffers on Si(001). The growth temperatures for the LT and HT layers were kept constant at 673 and 943 K, respectively, with no Ge growth occurring during the ramp between these temperatures, and with H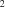 flowing inside the chamber. The temperature ramping rate was fixed at 4.5 counts per second. Post-growth *in situ* annealing was carried out on some wafers at 1103 K for 10 min in H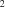. Different thicknesses for the LT and HT layers were achieved by varying the deposition times for each layer for growth on (111) and (110). The Ge growth rates at 673 K were determined to be approximately 0.05 nm s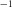 on (111) and 0.1 nm s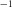 on (110); at 943 K these were 0.6 and 0.5 nm s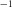. These growth rates are lower than the corresponding values on (100) of 0.3 and 1.5 nm s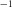 (Shah *et al.*, 2011 ▶). In the case of the (110) samples the thickness of the as-grown Ge epilayers was ∼420 nm, and in the (111) samples it was ∼500 nm. The thickness in each sample was controlled by cross-sectional transmission electron microscopy (TEM).

### 2.2. X-ray diffraction   

**Figure 1:**
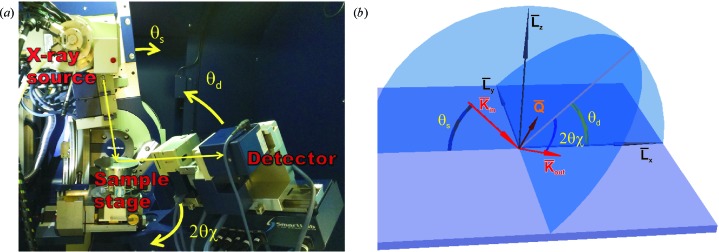
In-plane diffractometer. The angles are defined in the text. (*a*) The instrument configuration; (*b*) sketch of the wavevector arrangement.

Room-temperature measurements were performed using an in-plane-arm-equipped 9 kW SmartLab Rigaku diffractometer with a rotating anode providing Cu *K*α radiation (see Fig. 1 ▶). X-ray diffraction measurements were carried out in a parallel-beam geometry. A patented cross-beam optics unit was used for this purpose, which provides the parallel beam collimated vertically. A high-resolution setup with the combination of a four-crystal Ge monochromator in the 220 setting, a two-crystal Ge analyzer in the same setting and a scintillation counter was used to achieve sufficient resolution for the measurement of a set of samples.

In the case of the used diffractometer, the positions of the source and the detector can be described by the following instrumental angles (see Fig. 1 ▶):

(*a*) angle 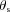, which is the angle between the line connecting the sample and X-ray source and the plane of the sample holder;

(*b*) angle 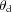, which is the angle between the axis of in-plane arm rotation and the plane of the sample holder; in the case of no in-plane arm rotation 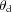 is the angle between the line connecting the sample and detector and the plane of the sample holder;

(*c*) angle 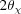, which is the angle of the in-plane arm rotation.

The angle 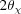 is specific to the model of in-plane diffractometer, and the measurement mode involving this additional detector movement degree of freedom will be considered below.

Sets of maps (in coplanar geometry, 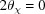) and scans (in noncoplanar geometry, 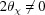) were measured for the two types of samples. The reciprocal space mapping was performed by a series of 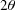
–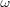 scans at various ω positions; the scintillation counter point detector was used. For the diffractometer used, the sample was not moved, the 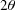 angle being 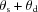 and the ω angle being 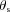. In the case of a (111)-oriented substrate, the sample was aligned in such a way that the 513 reflection was in the diffraction plane and the 513 and 153 RSMs were measured. Then the sample was aligned in such a way that the 242 reflection was in the diffraction plane and the 242, 333 and 404 RSMs were measured. The 333 RSM also was measured after rotation of the sample by 90°. In the case of an Si substrate with (011) orientation the sample was aligned in such a way that the 133 reflection was in the diffraction plane and the 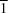
33 and 133 RSMs were measured; then the sample was aligned to the 026, 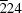 and 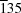 reflections and the 062, 242 and 153 RSMs were measured correspondingly.

**Figure 2:**
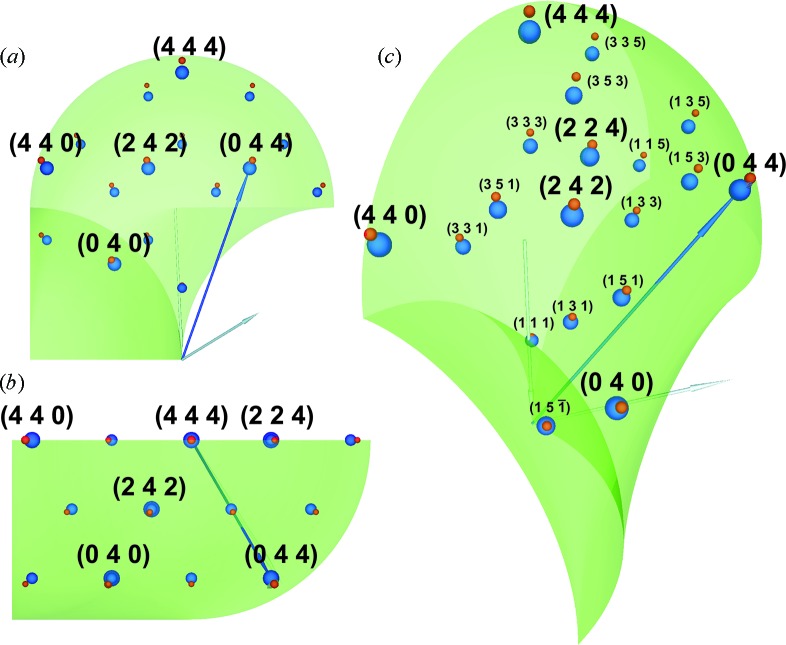
Sketch of incoming and outgoing beam arrangement for noncoplanar profile measurement of Ge reflection 044 on a (111)-oriented Si substrate. Red spheres denote the positions of Si Bragg reflections and blue spheres are related to the reflections of Ge. Green spheres limit the area with accessible points in reciprocal space when the sample is fixed. (*a*) corresponds to the side view, while (*b*) indicates the top view. (*c*) corresponds to a three-dimensional sketch of reciprocal space.

The in-plane movement of the diffractometer arm (the angle 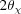) provides an additional degree of freedom in exploring the reciprocal space. A combination of 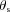, 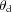 and 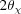 rotations enables us to put the transferred wavevector 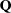 out of the conventional diffraction plane 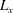 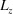 (see Fig. 1 ▶ *b*) and explore the reciprocal space without sample tilting. For the (111) substrate orientation, the sample was aligned in such a way that the 224 reflection was in the diffraction plane 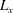 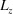. In this case 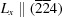. The 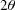
–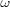 and 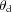 scans around the 040, 044, 133 and 242 reflections were measured. A sketch of the incoming and the outgoing beam arrangements for the noncoplanar profile measurement of reflection 044 is presented in Fig. 2 ▶. For the (011) substrate orientation, the sample was aligned in such a way that 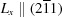; the scans around the 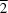
02, 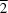
24, 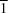
13, 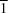
33 and 026 reflections were measured.

## 3. Diffracted X-ray intensity distribution   

### 3.1. General expressions for X-ray intensity distribution in reciprocal space   

The distribution of the diffracted (diffuse scattering) intensity from a crystal with defects in the reciprocal space is given by the Fourier transform of the correlation function *G* (Krivoglaz, 1996 ▶; Kaganer *et al.*, 2006*b* ▶; Benediktovitch, Feranchuk & Ulyanenkov, 2014 ▶): 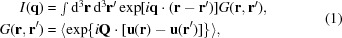
where 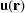 is the displacement at the site **r** due to randomly distributed dislocations, 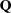 is the scattering vector, 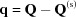 is the deviation of the scattering wavevector from reciprocal-lattice point 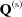, and the average 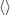 is performed over the dislocation positions. In the case of an epitaxial film 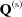 corresponds to the pseudomorphic strained film on the substrate.

We will focus below on almost completely relaxed films. In this case the film’s crystalline lattice is strongly distorted. Quantitatively, we will consider the case when 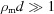, where 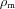 is the misfit dislocation density and *d* is the film thickness. Also, we will analyze the vicinity of the Bragg peak where most of the scattered intensity is concentrated; quantitatively, we will consider the case of 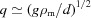. Under these assumptions the correlation between atomic positions drops off quickly and the main contribution to the scattered signal comes from closely spaced points centered far from dislocation lines, *i.e.* in the crystal areas that are most weakly distorted. In this case we can assume 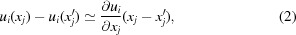
which considerably simplifies the calculations.

To calculate the correlation function 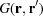 one has to know the displacement fields from a single defect. Below we will consider two types of defects: misfit dislocations and threading dislocations. In the case of misfit dislocations the displacement field is given in a coordinate system associated with the direction of the dislocation line 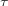 and sample normal 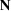. Let us denote this system Dm and define the direction of its axis as 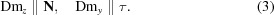
In this coordinate system the displacement field at the point 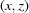 due to the dislocation line passing through the point 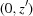 is expressed as 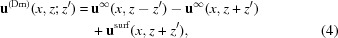
where 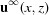 is the displacement field in the infinite medium of a dislocation at the origin, the first two terms on the right-hand side of equation (4) correspond to the dislocation itself and the image with respect to the surface, and the third term is the remaining surface relaxation. The explicit expressions for all these terms and for all Burgers vector orientations are given by, for example, Kaganer *et al.* (1997 ▶).

Performing the averaging over dislocation positions following the method outlined by Krivoglaz (1996 ▶) and Kaganer *et al.* (1997 ▶), and in the frame of approximation (2), the correlation function of the displacement fields of defects results in 
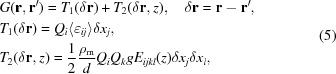
where 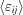 is the tensor of mean strain due to misfit dislocations, which is given by (in the Dm coordinate system) 
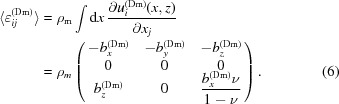
Here *b* is the Burgers vector of the dislocation and ν is the Poisson ratio. The tensor *E* in equation (5) is a fourth-rank tensor describing strain fluctuation. In analogy to the approach used in powder X-ray diffraction, this tensor is the elastic component of the dislocation contrast factor (Klimanek *et al.*, 1988 ▶; Martinez-Garcia *et al.*, 2009 ▶). In the case of epitaxial layers, this tensor becomes *z* dependent (which is not the case for powder X-ray diffraction), its components in the Dm coordinate system being equal to 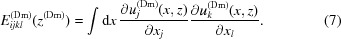
The integral over d*x* can be calculated analytically; the explicit expressions are given in Appendix *A* [App appa]. At large dislocation densities the elastic interaction between dislocations leads to spatial correlation between dislocation positions (Freund & Suresh, 2004 ▶). This positional correlation within the validity of approximation (2) leads to factor *g* in equation (5), which has the meaning of the ratio of the dispersion of distances between dislocation lines divided by the square of the average distance [see detailed discussion by Kaganer & Sabelfeld (2011 ▶)].

The second type of defect that will be important for us is threading dislocations running through the layer. Below, the threading dislocations are considered to follow the direction of the Burgers vector to maximize their screw nature (Bolkhovityanov & Sokolov, 2012 ▶). The expressions below are derived for screw threading dislocations; however, the tensor-based formalism presented here is general. Let us introduce the coordinate system Dt associated with the direction of the threading dislocation line 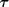: the *z* axis of the Dt system is directed along 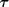; the directions of the *x* and *y* axes can be chosen arbitrarily in the plane normal to 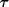: 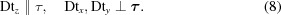
The correlation function becomes 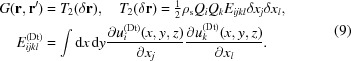
Here ρ_s_ is the threading dislocation density. To calculate the dislocation contrast elastic tensor *E* one has to know the displacement from a threading dislocation in the half-space and to calculate the two-dimensional integral over dislocation positions. The elastic displacement fields from an inclined dislocation in isotropic half-space were found by Yoffe (1961 ▶); the explicit expressions with corrected misprints are given by Shaibani & Hazzledine (1981 ▶). For this displacement field one can find that the displacement field derivative depends on the coordinates like 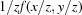. Hence from equation (9) it follows that the tensor *E* does not depend on *z*, *i.e.* it is constant within the layer. Besides the actual form of the displacement field the value of tensor *E* depends on how the two-dimensional integral is calculated. Since the displacement field derivative has an asymptotic behavior like 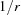, the integral is logarithmically divergent at both lower and upper limits. The truncation at the lower limit is done at a length scale corresponding to the termination of the validity range of the assumption of equation (2), while the truncation at the upper limit is done at a length scale corresponding to the dislocation correlation length (Kaganer & Sabelfeld, 2010 ▶; Benediktovitch, Feranchuk & Ulyanenkov, 2014 ▶). Both length scales are quite ill defined, but since the actual value of the tensor *E* depends on them logarithmicaly their influence is weak. Because of this fact we will neglect fine effects due to modification of the displacement fields related to the presence of the boundary and will use the solution for an infinite medium. In papers by Kopp and co-workers (Kopp, Kaganer, Baidakova *et al.*, 2014 ▶; Kopp, Kaganer, Jenichen & Brandt, 2014 ▶) the effect of boundary terms on the X-ray diffraction profile was accounted for using the direct assumption-free Monte Carlo approach and it was shown to be subtle. For the mentioned reasons we will use the displacement fields for an infinite medium, 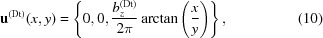
which results in the following nonzero components: 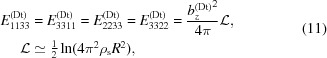
Here the logarithmic term 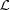 that appeared as a result of normalization is estimated according to Kaganer & Sabelfeld (2014 ▶). *R* is the correlation length of dislocation positions.

### 3.2. Measured X-ray intensity distribution   

The expressions in the previous section provide the intensity distribution 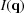 in three dimensions in reciprocal space. To experimentally access this distribution one would need a resolution function confined in three dimensions in reciprocal space, which is not the case in most of the used measurement modes. The recorded intensity and the intensity distribution in reciprocal space are connected by the resolution function 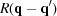: 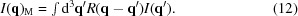
In commonly used measurement modes the resolution function does not provide the resolution in one or two directions. Accounting for this fact is crucial for processing the diffraction data. Several cases will be considered below.

With the help of the instrumental angles 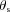, 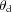, 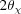 it is convenient to get the components of the wavevectors in the coordinate system associated with the sample stage and X-ray source. Let us denote this laboratory coordinate system as L and define the directions of its axes as 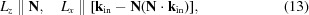
where 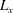 is parallel to the projection of the incoming vector on the sample surface. As one can see from equations (5) and (6), we will have need to perform convolutions of the vector 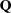 defined in the L system with the tensor *E* defined in the Dm or Dt system. To do this one has to transform both quantities to the same coordinate system. This can be easily done for the particular cases described by Kaganer *et al.* (1997 ▶) and Kopp, Kaganer, Baidakova *et al.* (2014 ▶), but for a general case one would need a universal recipe. In order to provide it, let us consider the crystallographic coordinate system C: 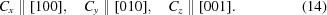
Here a cubic system has been considered for simplicity. The vectors 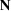 and **τ** are needed to define the Dm/Dt system. The direction of the incoming beam 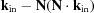 is known in the C system and is usually expressed in Miller indexes. The matrix 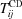 that transforms the components from the C system to the D system is easily obtained: its rows are the components of vectors 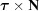, 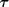, 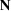 written in the C system. In a similar way one can obtain the elements of 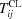. The needed matrix 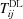 is obtained as 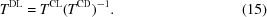



#### 3.2.1. Reciprocal space mapping   

Let us consider a triple crystal arrangement with a monochromator and an analyzer and the coplanar arrangement with 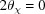. We assume that the resolution function in the diffraction plane is much narrower than the peak width due to the dislocation broadening, but in the direction normal to the diffraction the beam is not well conditioned and the resolution function is much wider than the peak. In this case equation (12) transforms to integration of 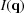 in the 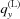 direction. Performing this integration in the expression (1) one gets the δ function in 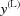, and for the measured intensity distribution 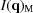 one obtains 
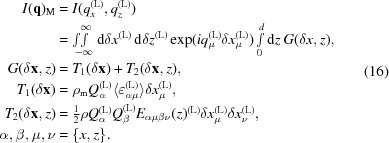
Here ρ stands for 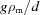 for the misfit dislocations and 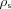 for threading dislocations. The term 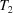 can be written in the form 
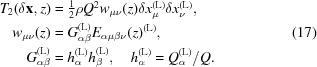
Here a 2 × 2 reflection-dependent matrix 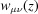 determines the shape of the peak. It has two contributions: the elastic due to the tensor *E* and the geometric due to the tensor *G*, which describes the dependence on the used reflection. Comparing to the similar expressions used in the powder diffraction case (Klimanek *et al.*, 1988 ▶; Martinez-Garcia *et al.*, 2009 ▶; Ungár *et al.*, 2001 ▶; Scardi & Leoni, 2002 ▶) one can see that 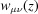 is the analog of the dislocation contrast factor, but it has two significant distinctions: (i) it is not a factor but a 2 × 2 matrix and (ii) in the case of misfit dislocations it is *z* dependent owing to the effect of the boundary.

#### 3.2.2. Noncoplanar out-of-plane scans   

The in-plane degree of freedom of a detector described by the angle 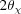 is favorable for the analysis of thin films (Ofuji *et al.*, 2002 ▶; Yoshida *et al.*, 2007 ▶), for texture analysis (Nagao & Kagami, 2011 ▶), for residual stress gradient investigation (Benediktovitch, Ulyanenkova, Keckes & Ulyanenkov, 2014 ▶) and for other applications. In the case of the epitaxial films studied here, the in-plane arm movement enables us to explore reciprocal space without sample rotation. Now we consider a measurement performed with a monochromator and an analyzer at 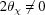. We will assume that compared to the peak width there is a large divergence of the source in the horizontal direction and a large acceptance of the detector in the 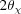 direction. In this case the measured signal is given by the integral of intensity in reciprocal space over the plane normal to the vector: 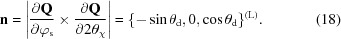
Here the components in the L coordinate system were calculated on the basis of the expressions for the scattering vector 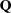 given by Benediktovitch, Ulyanenkova & Ulyanenkov (2014 ▶) and Benediktovitch, Ulyanenkova, Keckes & Ulyanenkov (2014 ▶). ϕ_s_ is the angle between the vector **k**_in_ and plane *L_x_L_z_* (see Fig. 1 ▶); it describes the divergence of the source in the horizontal direction. The integration over this plane results in 
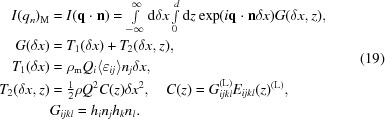
Here the quantity 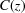 is again an analog of the dislocation contrast factor. As one can see from equations (17) and (19), the influence of the measurement mode on the measured intensity distribution is encapsulated by the geometric tensor *G*. Just for the illustration of this method of description let us consider several more measurement modes in terms of the geometric tensor *G*.

#### 3.2.3. Coplanar double-crystal scans   

In the case of the absence of an analyzer the measured intensity is integrated over the Ewald sphere, which can be approximated as a plane normal to the outgoing wavevector 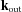 (Kaganer *et al.*, 2006*b* ▶, 2005 ▶). In this case for the intensity distribution one can use equation (19) with the vector 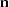 replaced by 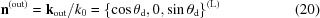
and the geometrical tensor 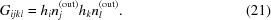



#### 3.2.4. Powder diffraction   

In the powder case, the intensity distribution provided by equation (1) corresponds to the intensity from a single grain, and the measured signal comes from grains with the different orientations. Because of this one has to integrate the intensity in reciprocal space over the plane normal to the transferred wavevector 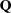 (Kaganer *et al.*, 2005 ▶; Benediktovitch, Feranchuk & Ulyanenkov, 2014 ▶). Hence for the geometrical tensor one obtains (Klimanek *et al.*, 1988 ▶) 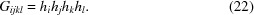
The elastic part *E* of the dislocation contrast factor depends on the dislocation type. By measuring various reflections and/or changing the measurement mode one will get the different geometric tensor *G* and hence the values of different combinations of the elements of the elastic tensor *E*. This way of obtaining information about the dislocation type was successfully demonstrated for a number of polycrystalline samples (Ungar, 2004 ▶; Ungár, 2001 ▶; Leoni *et al.*, 2007 ▶). Below we will try to adopt this approach to the characterization of the dislocation structure in Ge epitaxial layers.

## 4. Data processing and analysis   

**Figure 3:**
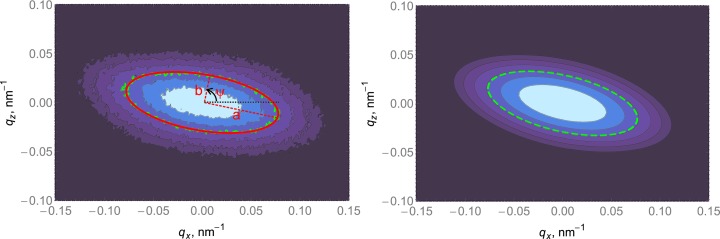
An example of a measured RSM of the 242 reflection, characterized by parameters 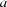, 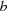, 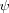 (left), and results of theoretical calculations (right). The dashed green line is the isointensity contour at the 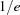 level, while the red solid line corresponds to fitting by an ellipse with the parameters 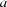, 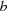, 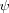. The intensity between the isocontours changes by a factor of 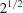.

With the help of equations (16)–(19) [Disp-formula fd18] [Disp-formula fd19] one can simulate the measured intensity distribution. We will consider the area close to the peak, where the approximation (2) holds. The results of calculations after equations (16)–(19) [Disp-formula fd18] [Disp-formula fd19] and experimental data show that the shape of the RSM is close to a two-dimensional Gaussian, and the shape of the profile is close to a Gaussian curve as long as the deviations from the peak center are of the order of 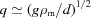 (see Figs. 3 ▶ and 4 ▶ for examples of measured data; other data look similar). At larger deviations from the peak center the shape transforms to a power law (Kaganer *et al.*, 2006*a* ▶). However, we will not use this low-intensity region in further analysis. Some asymmetry in the measured profile not predicted by the current formalism may be due to other defect types causing the peak broadening.

Within the considered approach the shape of all RSMs and profiles is determined by a small number of parameters, namely 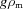 and 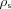. To process the data we propose to use a simple and illustrative approach close to a modification of the Williamson–Hall plot, which is intensively used in microstructure analysis of powder and polycrystalline samples (Ungár, 2001 ▶) [Shalimov *et al.* (2007 ▶) take a similar approach for application to heteroepitaxial film analysis]. The basis of the approach is to analyze the peak width dependence on the reflection. To assign a peak width to the data we will describe the profile by a single parameter *w*, which is the half-width at the 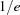 level, and in the case of the RSM three parameters *a*, *b*, ψ were used to describe the elliptic isointensity contour at the 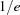 level (see Fig. 3 ▶).

### 4.1. Ge/Si(111)   

**Figure 5:**
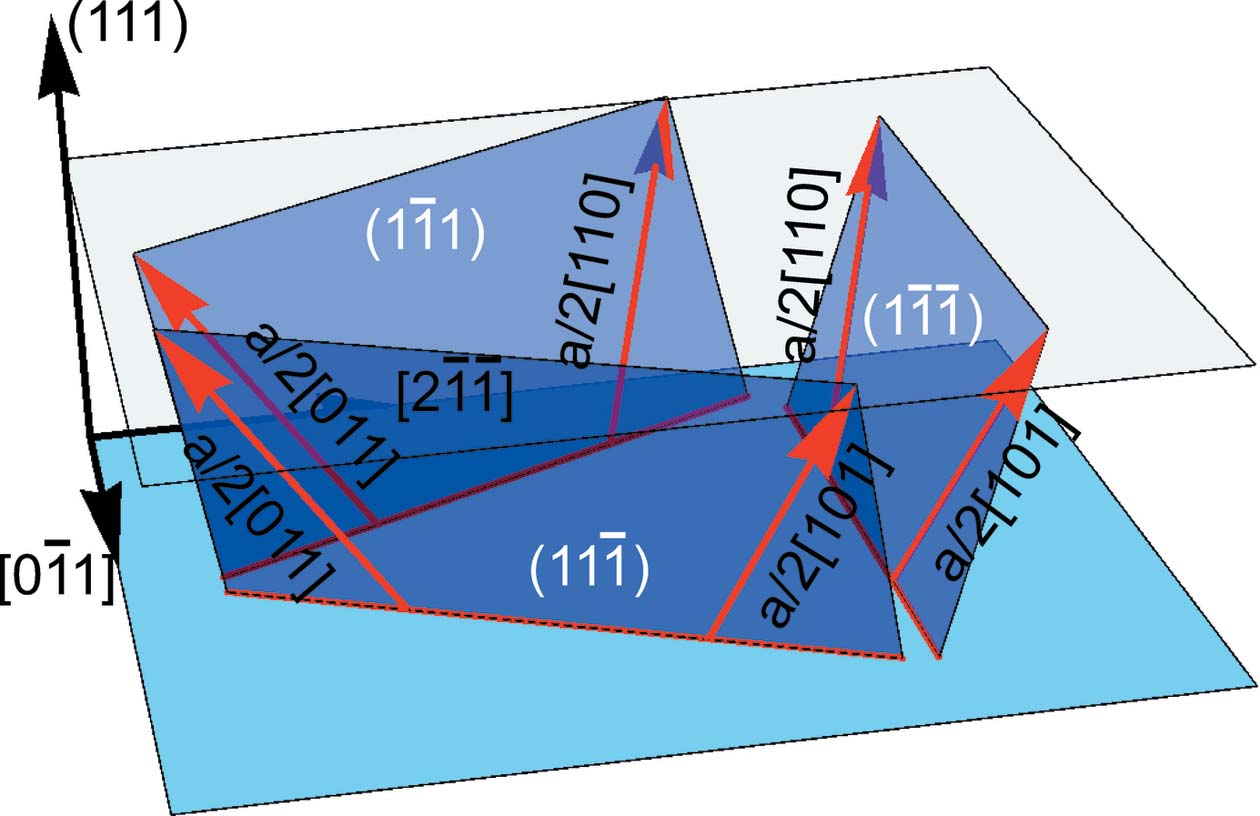
Dislocation configuration in Ge/Si(111) used for calculations.

For the Ge layer on the (111)-oriented Si substrate we have considered the dislocation configuration shown in Fig. 5 ▶. Three {111} slip planes are considered to be equally populated with, in total, six types of 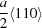 Burgers vector orientation (Nguyen, 2012 ▶). Assuming that all six misfit dislocation types have an equal dislocation density 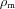 and are independent of each other, with the help of equation (6) transformed from the Dm to the L coordinate system we obtain for the average deformation 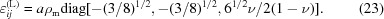
From the peak positions on the RSMs one can find that the Ge layer is completely relaxed. In order to compensate the mismatch between the Ge layer and Si substrate from equation (23), the necessary density of misfit dislocations is found as 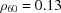
 nm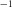. At such values of the dislocation density the coherent scattering is almost completely suppressed, and hence the observed diffraction signal is of diffuse origin, corresponding to the formalism presented above. The peak broadening due to dislocations is much higher than that due to the finite film thickness, and for this reason no thickness fringes are observed. The broadening due to the instrumental function effect was estimated by the substrate peak broadening, which transpired to be much narrower than the observed peaks and was omitted in the calculations.

The expression for the quantity 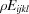 in the case of the considered dislocation system becomes 
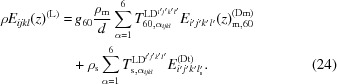
Here 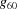 is a parameter for the positional correlation of misfit 60° dislocations, the index α denotes the dislocation type, 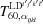 is a combination of four transformation matrices calculated for each misfit dislocation type α, 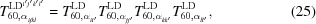
and 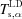 is the corresponding quantity for threading screw dislocations. The parameters 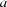, 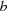, 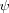, 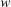 for the measured RSMs and profiles were calculated for the parameter 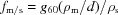 in the range 0–1, which is enough to find the parameters 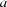, 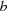, 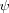, 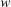 for any dislocation densities by simple scaling following from equation (24). The fitting of 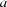, 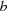, 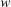 found from the measured data resulted in 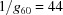, 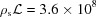
 cm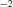. Since equation (11) and subsequent equations only contain the product 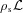 one cannot find 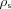 without the knowledge of *R*. However, the dependence on *R* is logarithmic and hence has only a weak influence on the result. We will further assume a typical value of *R* according to Kaganer & Sabelfeld (2014 ▶) and take 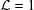. The misfit dislocations are strongly positionally correlated, which is expected for such a thick layer with a high mismatch. The density of threading dislocations qualitatively agrees with the value 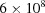
 cm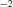 obtained by Nguyen (2012 ▶) by TEM for a similar sample.

**Figure 6:**
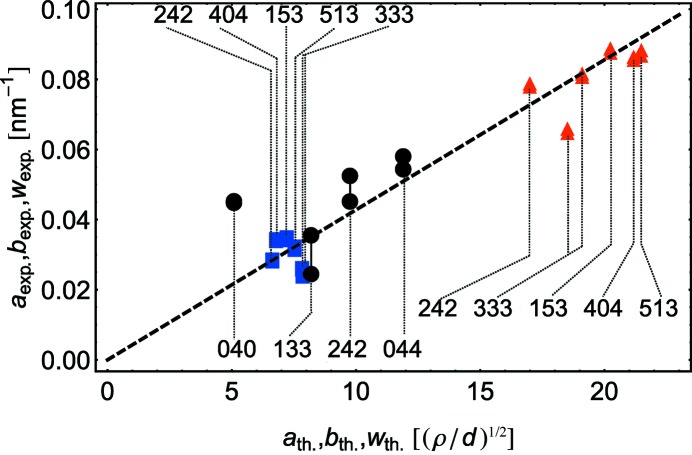
The experimentally measured peak width parameters *versus* calculated ones for parameters obtained by fitting (see discussion in text). Blue squares correspond to the value of parameter *a*, red triangles to *b* and black circles to *w*. Vertical lines join the points corresponding to measurements done at the same *hkl*. In the case of noncoplanar measurements the joined points correspond to the results of ω and 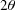
–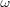 scans; in the case of RSMs they correspond to measurements in ‘+’ (large incidence and small exit angles) and ‘−’ (small incidence and large exit angles) geometries that almost coincide.

Fig. 6 ▶ plots the experimentally measured peak width parameters (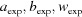) *versus* calculated ones (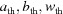) corresponding to the value of 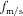 found from the fit and the value of 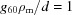. This plot can be considered as a modified Williamson–Hall plot (Ungár, 2001 ▶) adapted for thin-film analysis. In the ideal case all points should fall on the same line; at the given 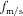 the slope of this line gives the absolute value of 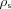 or 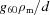. The intersection of this line with the ordinate axis gives the broadening due to the crystallite size and instrumental effects. In our case this contribution to the broadening is negligible owing to the large film thickness, which supports the assumptions made above.

### 4.2. Ge/Si(011)   

**Figure 7:**
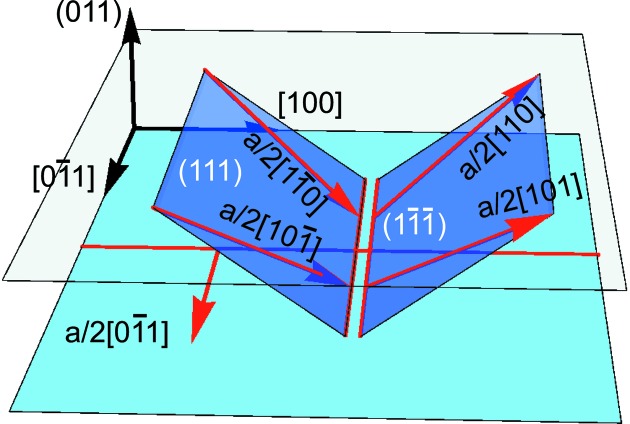
Dislocation configuration in Ge/Si(011) used for calculations.

A similar approach was applied for characterization of the Ge layer on the (011)-oriented Si substrate. The peak positions showed that this layer was also completely relaxed. However, the 60° dislocations are able to provide the relaxation only in one direction (Elfving *et al.*, 2006 ▶). For relaxation in the orthogonal direction we considered 90° dislocations. The resulting dislocation configuration is shown in Fig. 7 ▶. For the considered set of the four 60° misfit dislocations with the Burgers vector 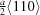 on {111} slip planes, a 90° dislocation and four threading dislocations that are considered to follow the direction of the Burgers vector, we obtain for an average deformation 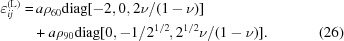
Here the setting 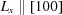 is considered. In order to compensate the mismatch between the Ge layer and Si substrate in all directions, the necessary density of the misfit dislocations is found from equation (26) as 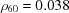
 nm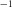 and 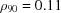
 nm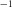.

The expression for the quantity 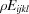 in the case of the considered dislocation system becomes 
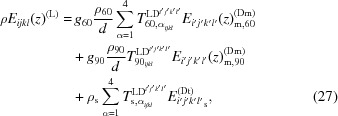
where 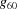 is a parameter for the positional correlation for the misfit 60° dislocations and 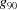 the equivalent for the 90° ones. The parameters 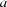, 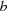, 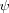, 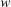 for the measured RSMs and the profiles were calculated for the parameters 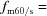 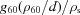, 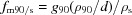 in the range defined by inequality 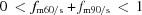, which is enough to find the parameters *a*, *b*, ψ, *w* for any dislocation densities by simple scaling following from equation (27). The fitting of *a*, *b*, *w* found from the measured data resulted in 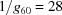, 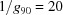, 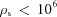
 cm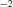. One can see that in this case the misfit dislocations are also strongly positionally correlated, which is expected for such a thick layer with a high mismatch. The density of the threading dislocations is underestimated. One of the reasons may be that we did not include the broadening due to the stacking faults (Huy Nguyen *et al.*, 2013 ▶). The incorporation of stacking faults into the current formalism will be the topic of future investigations.

**Figure 8:**
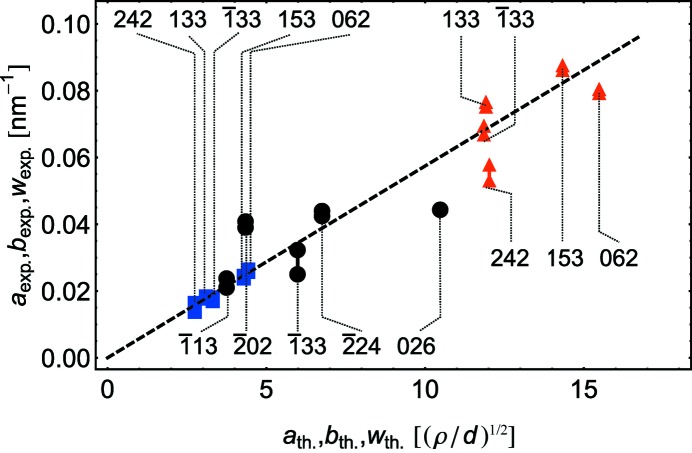
The experimentally measured peak width parameters *versus* calculated ones for parameters obtained by fitting. The same notation as in Fig. 6 ▶ is used.

The analog of the modified Williamson–Hall plot demonstrated in Fig. 8 ▶ shows that, similar to the Ge/Si(111) case, the contribution to the broadening due to finite film thickness is negligible.

## 5. Conclusions   

An approach to calculate the intensity distribution in reciprocal space in the vicinity of the Bragg peak due to arbitrary systems of straight misfit and threading dislocations at arbitrary sample normal orientation is formulated in a universal way, all necessary expressions being explicitly described. It is shown that the measured peak width is determined by the product of two tensors *E* and *G*, the first being determined by the strain fields produced by the defects and the second being dependent on the measurement mode only. Several examples of measurement modes are discussed in terms of the geometrical tensor *G*; the corresponding values of *G* are given in equations (17), (19), (21) and (22). The approach was applied for processing sets of RSMs and profiles measured in noncoplanar geometry for Ge/Si(111) and Ge/Si(011) layers. The measured intensity distributions were well described by Gaussians, which enabled us to use a small number of parameters associated with the shape and treat them in a manner similar to the modified Williamson–Hall plot. The misfit dislocations were found to be strongly positionally correlated, and the density of threading dislocations for the Ge/Si(111) layers were in qualitative agreement with TEM observations (Huy Nguyen *et al.*, 2013 ▶).

## Figures and Tables

**Figure 4:**
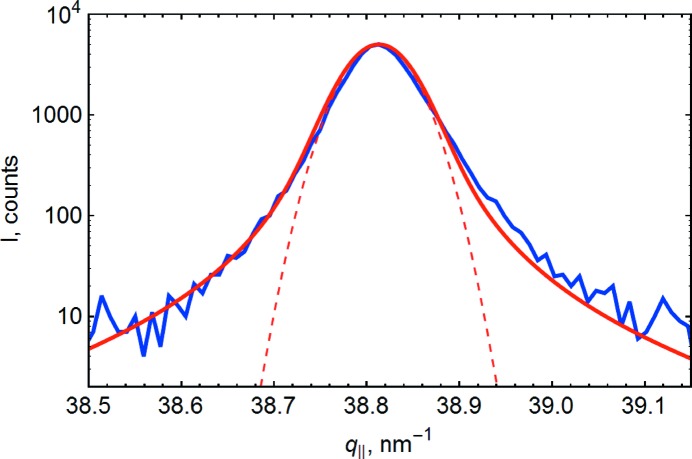
Scan of the 133 reflection converted to 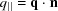 units. Red line: results of theoretical calculations; blue line: experimental data; thin dashed red line: approximation by Gaussian profile.

## Appendix A

The tensor  is a symmetric over permutation of the pair of indexes: . The nonzero components are given below:

Here ν is the Poisson ration, , ,  are the components of the Burgers vector, and all quantities are given in the Dm coordinate system. Its origin is taken at the free surface. The value of *z* in the above expressions is dimensionless and equal to the ratio , where *d* is the film thickness; hence  corresponds to the interface where misfit dislocations are lying.
